# Interface Engineering via Ti_3_C_2_T_*x*_ MXene Electrolyte Additive toward Dendrite-Free Zinc Deposition

**DOI:** 10.1007/s40820-021-00612-8

**Published:** 2021-03-08

**Authors:** Chuang Sun, Cuiping Wu, Xingxing Gu, Chao Wang, Qinghong Wang

**Affiliations:** 1 School of Chemistry and Materials Science, Jiangsu Normal University, Xuzhou, Jiangsu 221116 P. R. China; 2 Faculty of Engineering and Environment, Northumbria University, Newcastle upon Tyne, NE1 8ST UK; 3 Chongqing Key Laboratory of Catalysis and New Environmental Materials, College of Environment and Resources, Chongqing Technology and Business University, Chongqing, 400067 P. R. China

**Keywords:** Zinc metal batteries, Ti_3_C_2_T_*x*_ MXene, Electrolyte additive, Uniform Zn deposition

## Abstract

Zinc metal batteries have been considered as a promising candidate for next-generation batteries due to their high safety and low cost. However, their practical applications are severely hampered by the poor cyclability that caused by the undesired dendrite growth of metallic Zn. Herein, Ti_3_C_2_T_*x*_ MXene was first used as electrolyte additive to facilitate the uniform Zn deposition by controlling the nucleation and growth process of Zn. Such MXene additives can not only be absorbed on Zn foil to induce uniform initial Zn deposition via providing abundant zincophilic-O groups and subsequently participate in the formation of robust solid-electrolyte interface film, but also accelerate ion transportation by reducing the Zn^2+^ concentration gradient at the electrode/electrolyte interface. Consequently, MXene-containing electrolyte realizes dendrite-free Zn plating/striping with high Coulombic efficiency (99.7%) and superior reversibility (stably up to 1180 cycles). When applied in full cell, the Zn-V_2_O_5_ cell also delivers significantly improved cycling performances. This work provides a facile yet effective method for developing reversible zinc metal batteries.

**Supplementary Information:**

The online version contains supplementary material available at 10.1007/s40820-021-00612-8.

## Highlights

- Well-dispersed MXene nanosheets in the electrolyte dramatically shorten Zn^2+^ diffusion pathways and facilitate their migration.
- MXene interfacial layer with abundant functional groups and good conductivity induces uniform nucleation and enables long-term even deposition.
- MXene-containing electrolyte realizes dendrite-free Zn plating/striping with high Coulombic efficiency (99.7%) and superior reversibility (stably up to 1180 cycles).

**Supplementary Information:**

The online version contains supplementary material available at 10.1007/s40820-021-00612-8.

## Graphical abstract

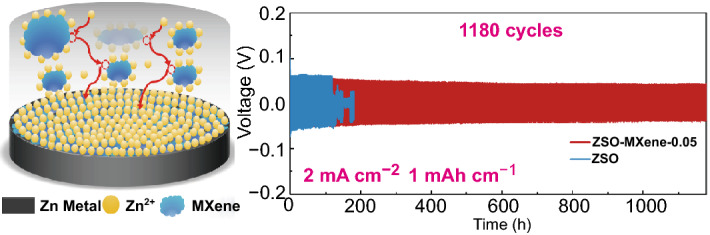

## Introduction

Lithium-ion batteries are dominant in the energy storage systems due to their high-energy density and long cycle life. However, the high cost, shortage of lithium resources and safety problem impede their large-scale applications [1–3]. Aqueous rechargeable secondary batteries, such as Zn-based, Al-based and Na/K-based systems, have become attractive alternatives due to the unique properties of the noninflammability and high ionic conductivity of aqueous electrolytes [4, 5]. Among various candidates, zinc metal batteries have attracted special attention since zinc metal anode possesses the inherent advantages of high theoretical specific capacity (820 mAh g^−1^), low redox potential (− 0.76 V, vs. SHE), as well as low cost, natural abundance and high safety [6–8]. Zn metal anodes enable the application of various cathodes for high-energy–density battery systems, e.g., Zn-air, Zn-MnO_2_ and Zn-V_2_O_5_ batteries [9, 10].

However, further development of Zn metal batteries is bottlenecked by the severe irreversible issue, which is caused by the inevitable dendrite formation under repeated Zn stripping/plating [11]. Zn dendrites may penetrate the separator and cause internal shorting, thus dramatically deteriorates the cycle life. Moreover, Zn dendrite exposes extra surface of fresh Zn metal and causes continuous sides reactions, resulting in electrochemically inactive “dead Zn,” thus decreases the Coulombic efficiency (CE) of Zn metal batteries [12]. Developing dendrite-free anode is critical for the further practical application of rechargeable Zn metal batteries.

To solve above-mentioned problems, various strategies have been attempted to regulate the nucleation and growth for suppressing the uncontrolled growth of dendrites. Typically, constructing nanostructured Zn anode [13] or high surface area Zn host, such as Zn-CNTs [14], Cu–Zn [15] and Zn@ZIF-8 [16] architectures, is proved to be effective for uniform Zn^2+^ deposition and alleviating the dendrite issue in neutral electrolyte. Besides, deliberately surface engineering on Zn metal is another effective approach to stabilize metal Zn anode. Nanoporous-CaCO_3_ [17], nano-TiO_2_ [18], polyamide [19], metal–organic framework [20] and porous Kaolin [21] have been reported to achieve uniform Zn^2+^ stripping/plating. However, they often suffer from complicated and precise controlling process, leading to consistency and cost issues for commercial application. Alternatively, a more facile approach using electrolyte additives, such as polyacrylamide [22], Et_2_O [23], has been developed to stabilize the solid/electrolyte interphase (SEI) and depress the dendrite formation by forming electrostatic shield on Zn surface. Nevertheless, these buffer layers generated by electrolyte additives are poorly conductive, which may hamper the homogeneous nucleation of Zn ions. Hence, a dual functional electrolyte additive, combining with the conductive advantages to enable fast electronic transmission while retaining their function of constructing robust SEI for ion transport, is still urgently desirable for the safe application of metallic Zn metal batteries.

**Fig. 1:**
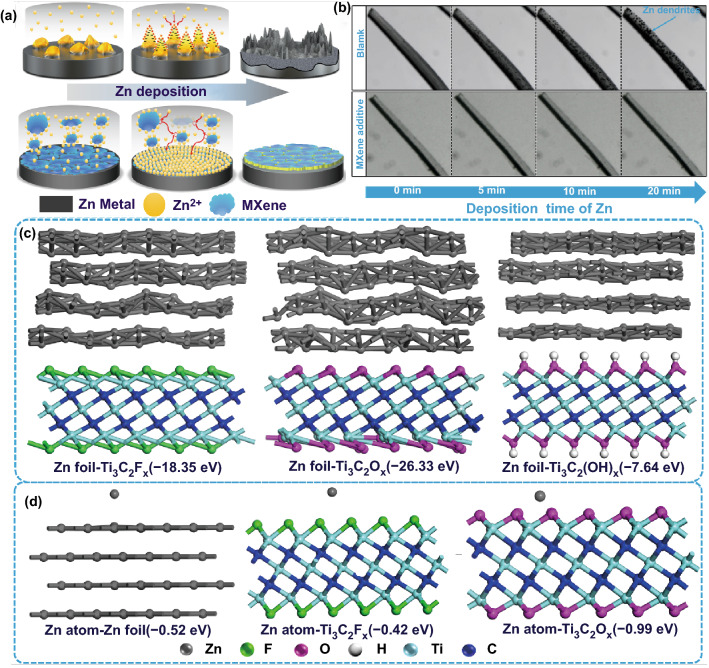
**a** Schematic illustration of the effect of MXene additive on the Zn deposition process. **b** In situ optical microscopy observation of Zn deposition at 4 mA cm^−2^ in blank ZnSO_4_ electrolyte and MXene-added electrolyte. Density functional theory calculation models of **c** Ti_3_C_2_T_*x*_ adsorbed on Zn foil and **d** Zn atoms absorbed on Zn foil, Ti_3_C_2_T_*x*_ and the corresponding binding energies

MXene is a new type of attractive 2D material, which possesses the unique properties of high surface area, abundant surface functional groups (-OH, -O, -F), metallic conductivity (up to 10^4^ S cm^−1^), strong hydrophilicity and good chemical stability [24–26]. Recently, various MXene-based architectures are proved to be ideal hosts for dendrite-free Li/Na/K plating/stripping [27–34]. Inspired by previous works, herein, in this work, Ti_3_C_2_T_*x*_ MXene was firstly proposed to be used as electrolyte additive to improve the irreversibility and kinetics of Zn plating/stripping. Massive Zn^2+^ and MXene additives can combine together via the electrostatic interaction and then they are adsorbed on the surface of Zn electrode to produce a conductive buffer layer, as illustrated in Fig. 1a. The MXene-Zn^2+^ functional layer can homogenize the dispersion of surface Zn^2+^ and provides well-dispersed “seed points” to induce uniform nucleation and homogenous ionic flux in the deposition process, inhibiting the growth of Zn dendrite. Moreover, MXene sheets in the electrolyte dramatically shorten Zn^2+^ diffusion pathways and facilitate their migration, thus the plating/striping kinetics will be improved. As a result, Zn metal anode delivers superior cycling stability and high CE using MXene-contained electrolyte regardless of in symmetric and full cells.

## Experimental Section

### Materials

The purity Zn foil is 99.99% and the thickness is 0.2 mm. Ti_3_C_2_T_*x*_ MXene nanosheets were supplied by Q-Changing Technology Co., Ltd (Zhengzhou, China). ZnSO_4_ was acquired from Sinopharm Chemical Reagent Co., Ltd. V_2_O_5_ was purchased from J&K Scientific Ltd. The spectator is Whatman Glass Fiber with the thickness of 90 mm.

### Characterization

The morphologies of MXene and zine deposition layer were conducted by a Hitachi SU-8010 SEM linked with EDS. XRD spectra were recorded using a Bruker-D8 ADVANCE X-ray diffractometer. Ionic conductivity of different electrolytes was performed by conductivity meter from Shanghai Shiyi Precision Instruments Co., Ltd. XPS was conducted on a Thermo Scientific K-Alpha + spectrometer equipped with a monochromatic Al Kα X-ray source (1486.6 eV) operating at 100 W. Renishaw inVia Raman systems (532 nm) were employed to analyze the compositions of the materials. Zeta Potential was measured by Malvern Zetasizer Nano S900. AFM measurements were carried out on BRUKER Dimension Icon. In situ optical microscopy observation was conducted on NIKON SMZ1270.

### Electrochemical Measurements

The electrolyte was 2 M ZnSO_4_ in DI water with/without Ti_3_C_2_T_*x*_ MXene nanosheets (0.02, 0.05, or 0.10 mg mL^−1^). Coin-type cells (CR2025) were assembled for Zn-Cu half cells, SS-SC, Zn-Zn symmetric cells and Zn-V_2_O_5_ full cells with the glass fiber as the separator. The amount of electrolyte was controlled at 90 μL. The ion conductivity of electrolyte was measured by AC impedance method in SS-SS symmetrical cells assembled with two stainless steel foils. The coulombic efficiency of Zn plating/striping on Cu foils was measured by stripping up to 1.0 V (vs. Zn^2+^/Zn). Linear polarization was measured by scanning with 1.0 mV s^−1^ between − 0.3 and 0.3 V. For the testing of Zn-V_2_O_5_ full cells, 1.0 g V_2_O_5_ was added into 15 mL NaCl aqueous solutions (2 M). After stirring for 72 h, the resulting precipitate was washed with deionized (DI) water and centrifuged and followed by drying overnight to obtain Na-V_2_O_5_ cathode material. V_2_O_5_ cathode was fabricated by compressing a mixture of Na-V_2_O_5_, Super-P and the binder polytetrafluoroethylene at a mass ratio of 7:2:1. Ethanol was used as the dispersant. The resultant paste was rolled into film and cut into pieces with areal mass loadings of 1.0 mg cm^−2^. The cut-off potentials of charge and discharge were set at 0.2 and 1.6 V (vs. Zn^2+^/Zn). Galvanostatic charge and discharge measurements were conducted on a LAND-CT2001A battery instrument (Wuhan, China) after resting for 4 h. Electrochemical impedance spectra (EIS, 10 MHz ~ 100 kHz) and cyclic voltammograms (CV, 0.2 mV S^−1^) measurements were taken on a CHI 604E electrochemistry workstation (Shanghai Chenhua Instrument, Inc).

### Computational Details

Density functional theory (DFT) calculations were performed using the generalized gradient approximation (GGA) [45] and Perdew–Burke–Ernzerhof (PBE) [46] exchange–correlation functional in DMol3 module of Materials Studio. Effective core potentials with DNP basis set and a DFT-D method within the TS scheme was employed during calculating. For the calculation of binding energy between MXene and Zn foil in aqueous electrolyte, the convergence tolerance was set to 1.0 × 10^−5^ Ha for energy, 2.0 × 10^−3^ Ha Å^−1^ for maximum force and 5.0 × 10^−3^ Å for maximum displacement. A vacuum thickness of 15 Å was applied to avoid interaction between the slab and its periodical images. The interface of Ti_3_C_2_T_*x*_ and Zn board was built by matching the 3 × 3 × 1 Ti_3_C_2_T_*x*_ and 4 × 4 × 2 Zn supercells. In the calculation of the combination between Ti_3_C_2_T_*x*_ and Zn board in aqueous ZnSO_4_, solvent environment was considered by using COSMO model with dielectric constant 78.54 of water.

For the calculation of binding energy between Zn atom and Ti_3_C_2_T_*x*_ MXene, the convergence tolerance was set to 1.0 × 10^−5^ Ha for energy, 2.0 × 10^−3^ Ha Å^−1^ for maximum force and 5.0 × 10^−3^ Å for maximum displacement. A k-point of 3 × 3 × 1 was selected to calculate adsorption energies of atomic zinc on a 3 × 3 Ti_3_C_2_T_*x*_ supercell. A vacuum thickness of 15 Å was applied to avoid interaction between the slab and its periodical images.

## Results and Discussion

### Characterization of MXene-containing Electrolyte

Delaminated Ti_3_C_2_T_*x*_ MXene nanosheets additives were obtained by selectively etching Al layers from MAX precursor (Ti_3_AlC_2_) in this work. X-ray diffraction (XRD) patterns shown in Fig. S1a confirms the fully transformation of Ti_3_AlC_2_ to Ti_3_C_2_T_*x*_ [35–37]. Scanning electron microscopy (SEM, Fig. S1d) and atomic force microscopy (AFM, Fig. S1b, c) images further reveal that the as-prepared MXene is of well-dispersed ultrathin sheet-like morphology with a thickness of ~ 1.5 nm. Elemental mapping results shown in Fig. S1e present uniform distribution of Ti, C, O and F species, confirming the existence of terminating functional groups of -O and -F. Due to the superior hydrophilicity and electrostatic repulsion between neighboring Ti_3_C_2_T_*x*_ MXene nanosheets, stable MXene colloid is formed in the ZnSO_4_ (ZSO) electrolyte, which is confirmed by Tyndall effect (Fig. S1f) [38, 39]. In order to explore the chemical stability of MXene in electrolyte, the phase transformation of MXene was characterized after soaking in the ZSO electrolyte for 72 h. As shown in Fig. S2, the crystal structure of MXene did not change. Moreover, after rested for 72 h, MXene nanosheets still well dispersed in the electrolytes (Fig. S3), confirming the superior chemical stability of MXene in the electrolyte. It is also vital to clarify the interaction between Zn^2+^ and MXene additive, thereby the MXene extracted from ZSO-MXene electrolyte has been characterized by XPS and Raman. As shown in Fig. S4, Zn–O bond can be detected for the MXene treated by ZSO electrolyte, indicating the strong interaction between Zn^2+^ and -O terminal groups of MXene, which is in good agreement with the DFT calculation. Raman spectrum shown in Fig. S5 reveals that the characteristic peaks of MXene still remains without obvious peak shift after treated by ZSO electrolyte, indicating the good stability of MXene in the electrolyte. Therefore, Zn^2+^ can be easily absorbed on the -O terminal groups of MXene, which can adjust ionic distribution at the interface. The good stability of modified electrolyte is helpful to ensure the long-term regulating function of MXene.

### Effect Mechanism of Ti_3_C_2_T_x_ MXene Additive on Zn Deposition

The effect of MXene additives on the initial Zn deposition process is illustrated via in situ optical microscopy. As shown in Fig. 1b, in blank ZSO electrolyte, uneven protuberances generate within 5 min plating and mossy Zn dendrites aggregate on the surface of Zn foil in 20 min at a current density of 4 mA cm^−2^. While in MXene-added electrolyte, the surface of Zn foil remains clean and free of dendrites in the whole process, indicating that the continuous growth of Zn dendrites is completely inhibited in the electrolyte with MXene additives.

**Fig. 2:**
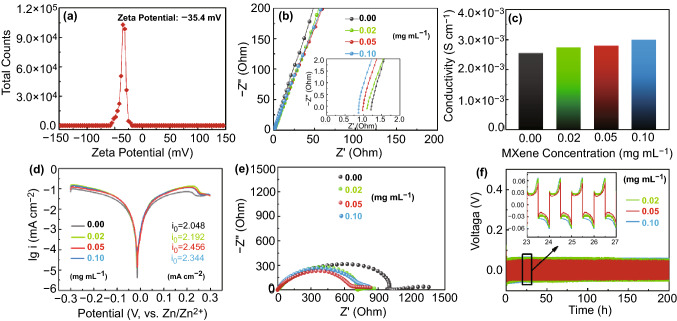
**a** Zeta potential distribution of the MXene nanosheets dispersed in water. **b** EIS of SS-SC cell in the electrolyte with different MXene concentrations. Insertion is the magnified curves of EIS at the high-frequency region. **c** Ionic conductivities of electrolytes with different MXene concentrations. **d** Linear polarization curves in the electrolyte with different MXene concentrations. **e** EIS of Zn-Zn symmetrical cell in the electrolyte with different MXene concentrations. **f** Galvanostatic cycling of Zn-Zn symmetrical cell at current density of 2 mA cm^−2^ with 1 mAh cm^−2^ platting/stripping

To investigate the effect mechanism of Ti_3_C_2_T_*x*_ MXene additive on Zn deposition, the interaction between Zn electrode and Ti_3_C_2_T_*x*_ MXene nanosheets in aqueous electrolyte was firstly investigated by DFT calculation. Considering the abundant functional groups, models of MXene with -OH, -F and -O terminal groups were constructed. As shown in Fig. 1c, the binding energy between -OH, -F and -O groups and metal Zn substrates is − 7.64, − 18.35 and − 26.33 eV, respectively, indicating strong adhesive tendency between MXene and Zn foil. Further experimental characterizations also verify such strong adhesive between MXene and Zn foil. Firstly, as shown in Fig. 2a, MXene nanosheets present the Zeta potential of -35.4 mV in water, demonstrating that they are prone to electrostatically interact with Zn metal. Then, a simple adsorption experiment was conducted to further confirm this interaction. As shown in Fig. S6a, Zn foil displays flat and clean surface in blank electrolyte. While an obvious MXene film is formed on Zn foil after immersed in MXene-added electrolytes for 4 h (Fig. S6b-d). And the MXene film is getting denser and denser with the increase of MXene concentration. It can be clearly seen that after soaking in ZSO-MXene-0.02, ZSO-MXene-0.05 and ZSO-MXene-0.1 electrolytes for 4 h, the thickness of the MXene interface are about 0.3, 0.5 and 1.0 µm, respectively (Fig. S7). Moreover, time-dependent experiment further reveals that MXene nanosheets were firstly flatted on Zn foil and then absorbed layer-by-layer to form a distinct electrode/electrolyte interface (Fig. S8).

Above results demonstrate that MXene nanosheets in ZSO electrolyte are perfectly absorbed on the surface of Zn foil and form an electrode/electrolyte interface. The Zn-philic properties of Zn foil and Ti_3_C_2_T_*x*_ MXene were further investigated by DFT to clarify the effects of MXene interface on Zn deposition. As shown in Fig. 1d, the binding energy between Zn atom and Ti_3_C_2_O_*x*_ (− 0.99 eV) is higher than those between Zn atom and Zn foil (− 0.52 eV), Zn atom and Ti_3_C_2_F_*x*_ (− 0.42 eV). Therefore, compared with Zn foil, MXene displays better affinity with atomic Zn via the -O group. The weak interaction between Zn atom and Zn substrate induces only a small amount of Zn nucleation and tends to result in Zn dendrites [40]. In contrast, in MXene-added electrolyte, the abundant zinc-oriented-O groups on the surface of MXene layer act as desired “seed points” for uniform nucleation and further induce even Zn deposition accompanying with fast electrons transfer, thus depressing the growth of Zn dendrites [9].

### Optimization of Ti_3_C_2_T_x_ MXene-Added Electrolyte

The effect of MXene concentration on the physical properties of electrolyte and Zn plating/stripping performance were investigated by preparing 0.02, 0.05 and 0.10 mg mL^−1^ MXene-containing electrolytes (named ZSO-MXene-0.02, ZSO-MXene-0.05, ZSO-MXene-0.1, respectively). Rapid ion transfer of electrolyte can effectively mitigate metal dendrite formation by forming homogeneous ionic flow, which can quickly reduce the ion concentration gradient at the solid/liquid interface [41, 42]. Therefore, the ionic transportation capability of MXene-added electrolyte is assessed by AC impedance method in Stainless Steel symmetric cells (SS-SC). As shown in Fig. 2b, the electrolyte resistance decreases with the increase of MXene concentration, thereby affording a higher ionic conductivity. Furthermore, ionic conductivity of different electrolytes was performed by conductivity meter. As shown in Fig. 2c, the ion conductivities of ZSO, ZSO-MXene-0.02, ZSO-MXene-0.05 and ZSO-MXene-1.0 are 2.56, 2.74, 2.81 and 3.01 mS cm^−1^, respectively, demonstrating that the ionic conductivity increases with the introduction of MXene. The significant improvement of Zn^2+^ conductivity could be attributed to the shortened Zn^2+^ transport pathway resulting from strong Zn^2+^ adsorption of MXene.

The kinetics is an important parameter for evaluate the properties of Zn metal batteries. Electrolyte also plays an import role on the kinetics of Zn deposition. Therefore, the relative electrochemical characterizations have been employed to evaluate the improved kinetics by adding the MXene additives. First, Tafel plots analysis shown in Fig. 2d reveals that the exchange current density increases from 2.048 (blank ZSO) to 2.456 (ZSO-MXene-0.05) mA cm^−2^, then slightly decrease to 2.344 mA cm^−2^ (ZSO-MXene-0.1), indicating enhanced Zn^2+^ diffusion by adding the MXene in the ZSO electrolyte [43, 44]. And the optimal additive concentration may be 0.05 mg mL^−1^. Then, the interfacial Zn^2+^ transport of Zn-Zn cell was investigated by conducting EIS measurements. As shown in Fig. 2e, all the Nyquist plots of Zn-Zn cells present similar curves including a semicircle in the high-frequency region that represents the interface charge-transfer resistance (*R*_ct_) and a line in the low-frequency region that stands for the diffusion resistance. Obviously, the cells with MXene additives deliver lower *R*_ct_ than blank ZSO electrolyte, demonstrating improved interfacial charge transport during the electrochemical reaction process [21, 22]. The enhancement is attributed to the accelerated Zn^2+^ diffusion in the electrode/electrolyte interface and enhanced charge transfer of MXene buffer layer. The equivalent circuit and fitting results are provided in Fig. S9 and Table S1. It is noted that the cell with ZSO-MXene-0.05 electrolyte possesses the highest exchange current density and lowest *R*_ct_ (715.2 Ω), demonstrating the best kinetics, which again proves the optimal additive concentration (0.05 mg mL^−1^).

Moreover, the Zn plating/striping properties in the electrolyte with different MXene concentration are investigated via assembling symmetric Zn-Zn cells. As shown in Fig. 2f, the Zn-Zn cells with MXene additives display good reversibility at a current density of 2 mA cm^−2^ with a constant capacity of 1 mAh cm^−2^. Insertion of Fig. 2f shows the comparison of voltage profiles in each electrolyte. It is clearly seen that the cell with 0.05 mg mL^−1^ MXene additives gives lowest overpotential of 50 mV. As MXene concentration increases to 0.1 mg mL^−1^, overpotential becomes higher. According to the above analysis, both too low and too high MXene additive concentration cannot further improve the electrochemical performances as the insufficient MXene in the electrolyte cannot form a complete and stable SEI layer, while excessive MXene in the electrolyte will produce much thicker SEI that possibly reduces the kinetics. Such plating/striping experimental results are also in good agreement with EIS results in Fig. 2b, e.

### Zn Plating/Striping Behavior in Optimized Electrolyte

**Fig. 3:**
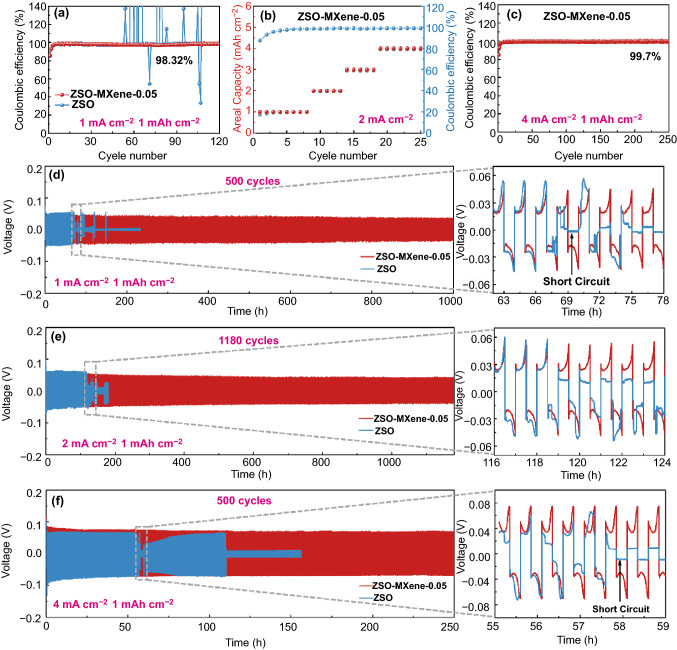
(**a**) Coulombic efficiency of the Zn plating/stripping at 1 mA cm^−2^ with the capacity of 1 mAh cm^−2^. **b** Coulombic efficiencies of the Zn plating/stripping at 2 mA cm^−2^ with the capacity of 1, 2, 3 and 4 mAh cm^−2^. **c** Coulombic efficiencies of the Zn plating/stripping at 4 mA cm^−2^ with the capacity of 1 mAh cm^−2^. **d-f** Long-term galvanostatic cycling of Zn-Zn symmetrical cell at 1, 2 and 4 mA cm^−2^ with the capacity of 1 mAh cm^−2^

Therefore, detailed Zn plating/striping behavior in optimized electrolyte (ZSO-MXene-0.05) was further investigated. CE is a critical indicator to judge the reversibility and practicality of Zn metal batteries. The CE of Zn plating/stripping on Cu foil with the plating time of 1 h and striping to 1.0 V at a current density of 1 mA cm^−2^ is presented in Fig. 3a. It is noted that the electrode presents relatively lower CE in the first four cycles in both of the electrolytes. It may be caused by the partial coverage of zinc by zinc-hydroxide or zinc-oxide in the initial cycles. In blank ZSO electrolyte, irregular fluctuation of CE is observed after only ~ 50 cycles, which can be indexed to the vigorous growth of dendrites on the surface of Zn substrate. In contrast, the Zn electrode exhibits steady high CE of 98.32% over 120 plating/stripping cycles in ZSO-MXene-0.05 electrolyte and when the current density increased to 2 mA cm^−2^ with step-increased areal capacities of 2, 3 and 4 mAh cm^−2^ (Fig. 3b), excellent reversibility still can be obtained (with high CE of 98.8% at 4 mAh cm^−2^). The high CE and stable cyclability in MXene-added electrolyte can be attributed to the MXene buffer layer absorbed on Zn foil, which prevents the formation of hydronium ion and relieves the side reactions of Zn in the concentrated zinc sulfate solution. The advantages using MXene-containing electrolyte can be further illustrated by its low initial overpotential. As shown in Fig. S10, both of the voltage profiles obtained at 1 mA cm^−2^ with the capacity of 1 mAh cm^−2^ in blank ZSO electrolyte and in ZSO-MXene-0.05 electrolyte present a voltage dip at the initial Zn plating process, which is corresponding to the nucleation process [29]. Obviously, the overpotential of Zn metal deposition is much smaller in ZSO-MXene-0.05 electrolyte (~ 27 mV) than that in ZSO electrolyte (~ 51 mV), indicating lower nucleation barriers with MXene additives. Such a result confirms the strong regulating ability of MXene surface layer during nucleation process. The advantages in ZSO-MXene-0.05 electrolyte can be further displayed by high CE of 99.7% at higher current density of 4 mA cm^−2^ (Fig. 3c).

The strong regulation of MXene-based buffer layer can contribute to a long-term cycling stability and the cycling curves verified in symmetric Zn-Zn cells are given in Fig. 3d-f. As shown, in blank ZSO electrolyte, the cell delivers approximate voltage polarization during cycling but finally short circuits at 35th and 124th cycles at the current density of 1 and 2 mA cm^−2^ with a fixed capacity of 1 mAh cm^−2^, which is caused by the formation of mossy Zn dendrite. Remarkably, the Zn-Zn cell in ZSO-MXene-0.05 electrolyte displays regular charge/discharge curves without obvious fluctuation and ultralong cycle life over 500 cycles at 1 mA cm^−2^ and 1100 cycles at 2 mA cm^−2^, indicating high stability of Zn foil. When the current density is increased to 4 mA cm^−2^ with the fixed capacity of 1 mAh cm^−2^ (Fig. 3f), a cycle life of more than 500 cycles is still obtained, which is much longer than that obtained in ZSO blank electrolyte (~ 100 cycles). Even when the plating capacity enhanced to 4 mAh cm^−2^, excellent cycle stability of 120 h still can be obtained at 4 mA cm^−2^ (Fig. S11). Such excellent cycling stability is far superior to the previous reports as shown in Table S2. The improvement of cycling stability further confirms that MXene additives significantly contribute to the suppression of dendrites growth. Moreover, the decrease in cycle life at higher current density may be caused by the easily formation of Zn dendrites at high current density, which is not benefit for the diffusion of Zn^2+^ at the electrode/electrolyte interface.

**Fig. 4:**
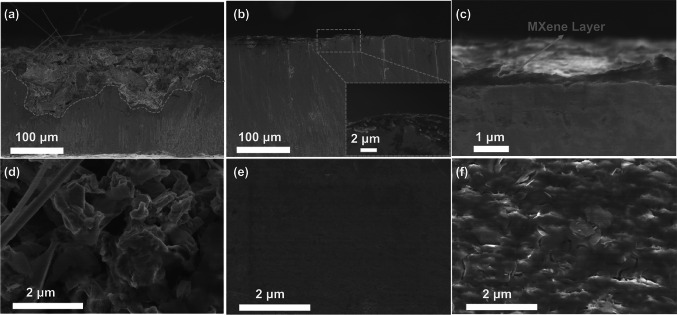
Cross-sectional and surface configuration of Zn anode after cycling at current density of 2 mA cm^−2^ with 1 mAh cm^−2^ Zn plating/stripping: **a, d** in ZSO electrolyte after 50 h, **b, e** in ZSO-MXene-0.05 electrolyte after 50 h and **c, f** 400 h

The inhibition of dendrite growth can be clearly illustrated by the SEM images of Zn electrode after cycling. As shown in Fig. 4a, d, Zn anode is seriously corroded from surface to depth in blank electrolyte. Massive dendritic Zn is obviously observed after 50 plating/stripping cycles at a current density of 2 mA cm^−2^. In contrast, in MXene-added electrolyte, Zn anode presents dense and even Zn deposition with no obvious surficial change after 50 cycles (Fig. 4b, e). Even after 400 cycles, flat MXene buffer layer with no protuberance is clearly observed (Fig. 4c, f).

Moreover, to evaluate the stability of MXene film during the cycling process, the cross-sectional, surface configuration and EDS mapping of Zn anode after cycling at current density of 2 mA cm^−2^ with 1 mAh cm^−2^ Zn plating/stripping were characterized. As shown in Figs. S12 and S13, even after 200 cycles, the MXene layer still closely absorbed on Zn film and the surface still keeps smooth, displaying good stability. And the corresponding EDS mapping results in Fig. S13 reveal the existence of Ti and Zn elements on the surface even after 200 cycles, demonstrating that MXene stably exists to participate in the formation of SEI film during Zn^+^ deposition. XRD measurement of cycled Zn anode in the ZSO and ZSO-MXene-0.05 electrolytes has been conducted after 3 cycles. As shown in the insertion of Fig. S14, the Zn anode after three cycles in blank ZSO electrolyte presents two obvious peaks, which can be indexed to (Zn(OH)_2_)_3_(ZnSO_4_)(H_2_O)_3_ (PDF#78–0247), indicating the formation of by-products. In contrast, the XRD pattern of Zn anode after 3 cycles in ZSO-MXene-0.05 electrolyte displays no peaks of by-products. Moreover, the peak of MXene can be clearly detected, indicating that MXene layer keeps stable during the cycling process and can effectively inhibit side reactions. In general, the high stability and long-term protecting effect of conductive MXene buffer layer helps to produce smooth Zn deposition and avoid the electrode destruction.

EIS measurements of the Zn-Zn cells in ZSO and ZSO-MXene-0.05 electrolyte after charge/discharged for 50 cycles were also conducted (Fig. S15). Obviously, *R*_ct_ of the anode in MXene-added electrolyte significantly decreased after Zn deposition compared to the fresh cells. It may due to the formation of heterogeneous seeded nucleation of Zn on MXene-coated surface and reversible dissolution, which also contributes to the improved reaction kinetics and cycling performance.

### Electrochemical Performance of Zn-V_2_O_5_ Full Cells Using MXene-containing Electrolyte

**Fig. 5:**
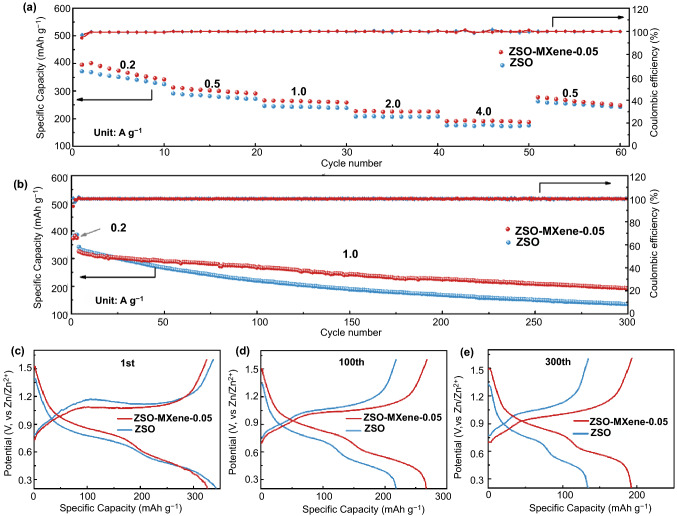
Electrochemical performance of full cells with and without MXene additive **a** Rate performance of Zn-V_2_O_5_ full cells from 0.2 to 4 A g^−1^. **b** Long-term cycling performance of Zn-V_2_O_5_ full cells. Voltage profiles of Zn-V_2_O_5_ full cells at current density of 1 A g^−1^ for the **c** 1st, **d** 100th and **e** 300th cycle

Above measurements illustrate that MXene as the additive could significantly contribute symmetric Zn-Zn cells to deliver high CE, good rate capability and ultralong cycle life. To further evaluate the practicality, Zn-V_2_O_5_ full cells employing MXene-containing electrolyte were fabricated and investigated. Cyclic voltammograms measurements of V_2_O_5_/Zn batteries in ZSO and ZSO-MXene-0.05 electrolyte were performed at a scan rate of 0.2 mV S^−1^ with the potential range of 0.2 ~ 1.6 V. As shown in Fig. S16, the two cells present similar shape with two pairs of redox peaks, which can be attributed to the two-step (de)intercalation process of Zn^2+^ during the charge/discharge processes. Note that compared with the cell using blank ZSO electrolyte, the anodic peaks of that in MXene-added electrolyte shift negatively (~ 128 mV) and the cathodic peaks shift positively (~ 93 mV), indicating smaller polarization [14]. The impact of MXene additive on the self-discharge behavior of Zn full cells are import to prove the valuable of this concept in practical use. Self-discharge behavior of Zn full cells in MXene-added electrolyte have been provided. It is found that the as-fabricated Zn-V_2_O_5_ full cell presents stable voltage without obvious self-discharge (Fig. S17). As shown in Fig. 5a, MXene-containing electrolyte enables Zn-V_2_O_5_ full cell with superior rate capability. The average specific capacities are 390.9, 310.6, 265.0, 227.5 and 190.5 mAh g^−1^ at current densities of 0.2, 0.5, 1.0, 2.0 and 4.0 A g^−1^, respectively, which are higher than those obtained in the blank ZSO electrolyte. When the current density was set back to 0.5 A g^−1^, the specific capacity still remains 277.3 mAh g^−1^, further indicating the good cycling reversibility under abusing testing condition. The advantages using MXene-contianing electrolyte can be more clearly illustrated via the comparison of long-term cycling curves (Fig. 5b). The Zn-V_2_O_5_ full cell using modified electrolyte delivers a high initial capacity of 326.4 mAh g^−1^ and high reversible capacity of 192.9 mAh g^−1^ after 300 cycles at the current density of 1.0 A g^−1^ with a steady high CE of approaching 100%. A high capacity retention of approximately 60% could achieve. Excitingly, this kind of Zn-V_2_O_5_ full cells with using MXene-added electrolyte illustrates better or competitive electrochemical performances compared to previous reported Zn-V_2_O_5_ full cell systems as shown in Table S3. While a rapid capacity fading (135 mAh g^−1^ after 300 cycles) and low capacity retention (39.4% after 300 cycles) are observed in blank ZSO electrolyte. Additionally, the galvanostatic profiles at different cycles obtained at current density of 1 A g^−1^ are shown in Fig. 5c-e, which further demonstrate that reduced voltage gaps and higher capacity retention of Zn-V_2_O_5_ cells using MXene-added electrolyte are superior to blank ZSO electrolyte. As shown in Fig. S18, the full cell with higher V_2_O_5_ mass loading of 5.8 mg cm^−2^ delivers high initial areal capacity of 2.32 mAh cm^−2^ at the current density of 1.16 mA cm^−2^, which still remains 1.35 mAh cm^−2^ after 80 cycles, demonstrating high specific capacity and good cycling stability. Therefore, using MXene as electrolyte additive could simultaneously demonstrate the coupling of conductivity advantages and the function of participating in the robust SEI formation and thus it is an effective way to construction of Zn dendrite-free system with better performance.

## Conclusions

In summary, an efficient design, constructing conductive and resilient surface layer on the surface of Zn anode, is proposed to prohibit the dendrite growth and easily realized via using MXene-contained electrolyte. The MXene-based buffer layer can well regulate the uniform nucleation of Zn^2+^ due to its good conductivity and strong binding energy with Zn^2+^ and well accommodate the surface structure change due to the excellent elasticity of well-dispersed MXene nanosheets. They synergistically suppress the Zn dendrites growth and relieve the side corrosions during the continuous plating/striping process. As a result, with the assistance of MXene additive, the Zn anode presents stable cycling performance of more than 1100 cycles at 2 mA cm^−2^, as well as high CE of nearly 100%. The as-fabricated Zn-V_2_O_5_ full cell with MXene-added electrolyte also displays high specific capacity (326.4 mAh g^−1^ at 1.0 A g^−1^), good rater capability (190.5 mAh g^−1^ at 4 A g^−1^) and long cycling stability (up to 300 cycles). This work provides a facile and low-cost strategy for developing dendrite-free metal-based batteries.

## Supplementary Information

Below is the link to the electronic supplementary material.

Supplementary file 1 (PDF 1842 kb)
