# Supplementary material for: Interface Engineering via Ti3C2Tx MXene Electrolyte Additive toward Dendrite-Free Zinc Deposition
Source: Nanomicro Lett. 2021 Mar 8;13:89. doi: 10.1007/s40820-021-00612-8 (PMC8006525; doi:10.1007/s40820-021-00612-8)
Supplement: Supplementary file 1 — (PDF 1842 kb) [file 40820_2021_612_MOESM1_ESM.pdf]

Supporting Information for

Interface Engineering via $Ti_3C_2T_x$ MXene Electrolyte Additive towards Dendrite-Free Zinc Deposition

Chuang Sun[1, +], Cuiping Wu[1, +], Xingxing Gu[2, 3,] *, Chao Wang[1,] *, Qinghong Wang[1,] *

[1] School of Chemistry and Materials Science, Jiangsu Normal University, Xuzhou, Jiangsu 221116, P. R. China

[2] Faculty of Engineering and Environment, Northumbria University, Newcastle upon Tyne NE1 8ST, UK

[3] Chongqing Key Laboratory of Catalysis and New Environmental Materials, College of Environment and Resources, Chongqing Technology and Business University, Chongqing 400067, P. R. China

[+]Chuang Sun and Cuiping Wu contributed equally to this work

*Corresponding authors. E-mail: x.gu@ctbu.edu.cn or xingxing.gu@northumbria.ac.uk (Xingxing Gu), wangc@jsnu.edu.cn (Chao Wang), wangqh@jsnu.edu.cn (Qinghong Wang)

Supplementary Figures and Tables

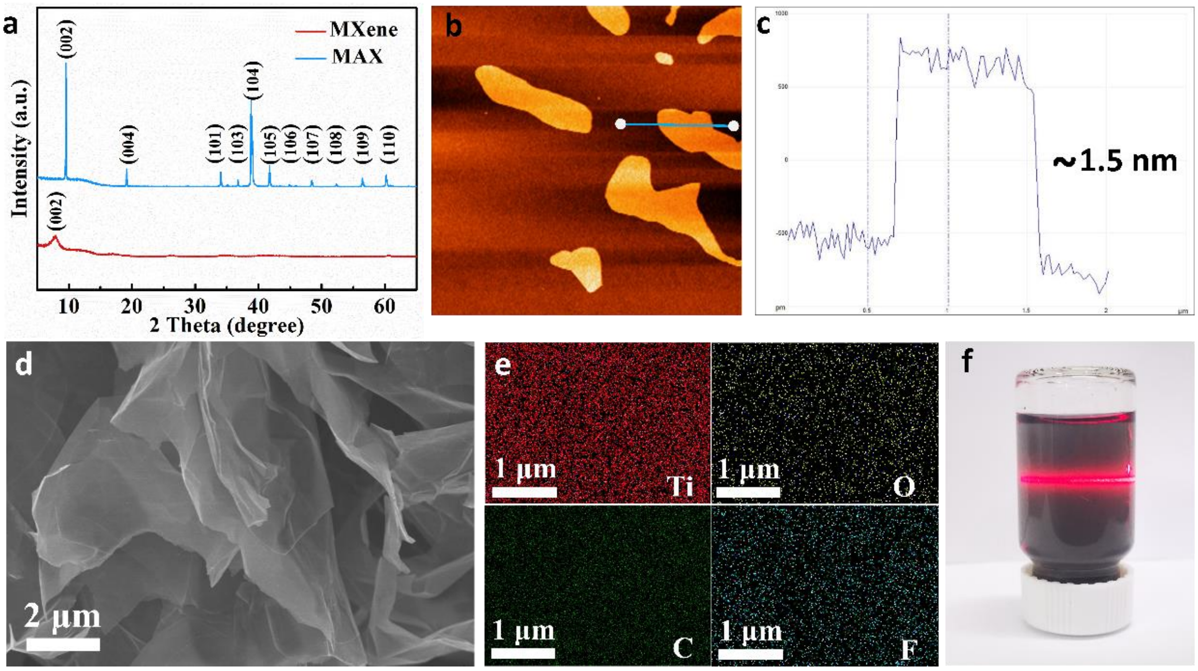

Fig. S1 Characterization of the as-prepared $Ti_3C_2T_x$ MXene: **a** XRD pattern, **b, c** AFM images, **d** SEM image, **e** EDS mapping and **f** Tyndall effect of MXene concentration of 0.05 mg mL^{-1} for electrolyte additive

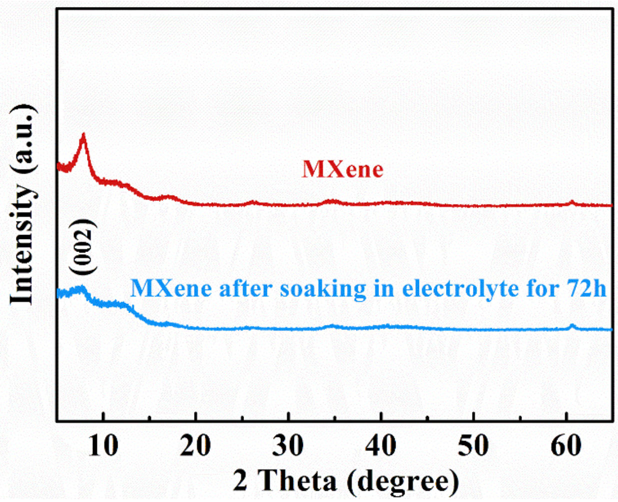

Fig. S2 XRD pattern of MXene before and after soaking in electrolyte for 72 h

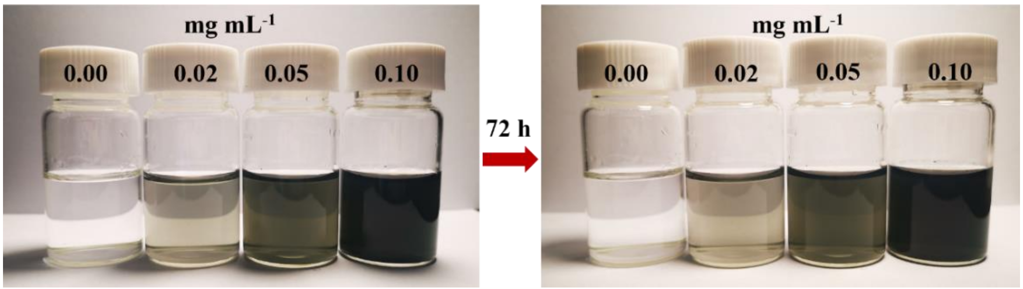

Fig. S3 Photos of the freshly prepared MXene added ZSO electrolytes and those rested for 72 h

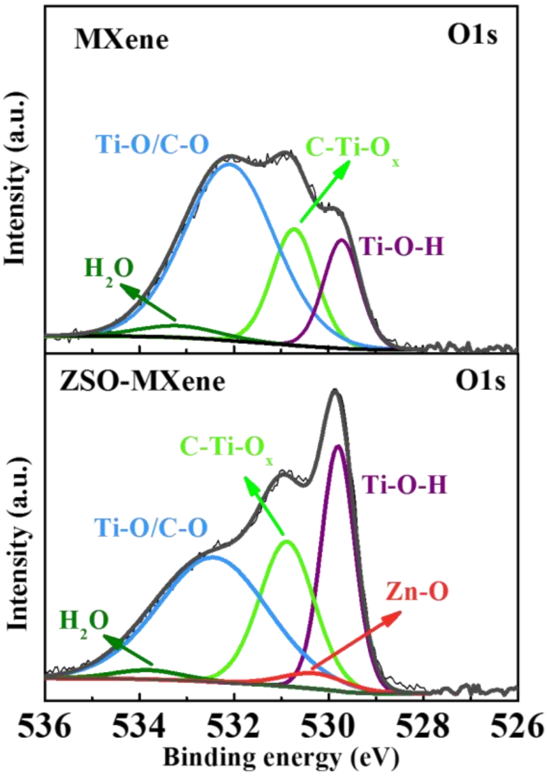

Fig. S4 Comparison of high resolution XPS spectra of pristine MXene and MXene after soaking in ZSO electrolyte in the O 1s region

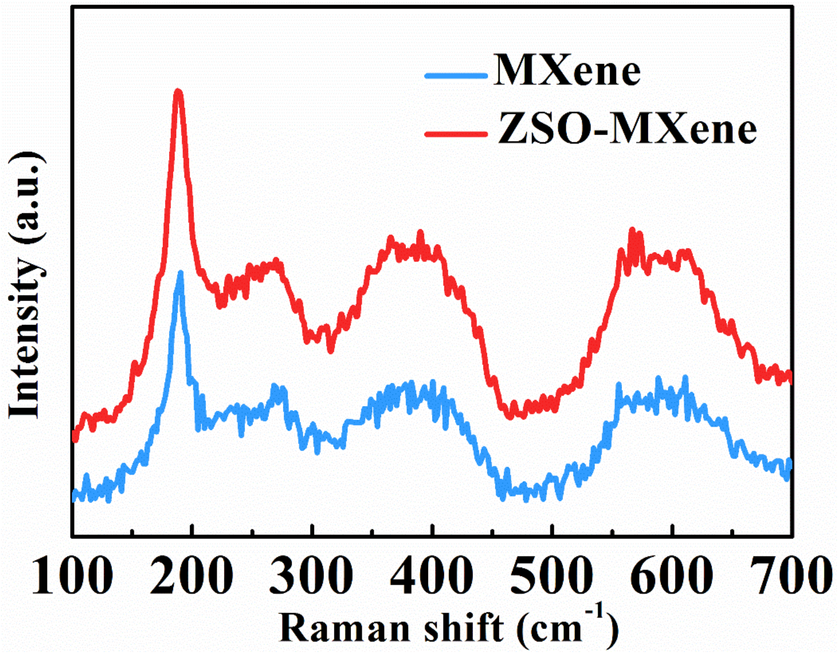

Fig. S5 Comparison of Raman of pristine MXene and MXene after soaking in ZSO electrolytein

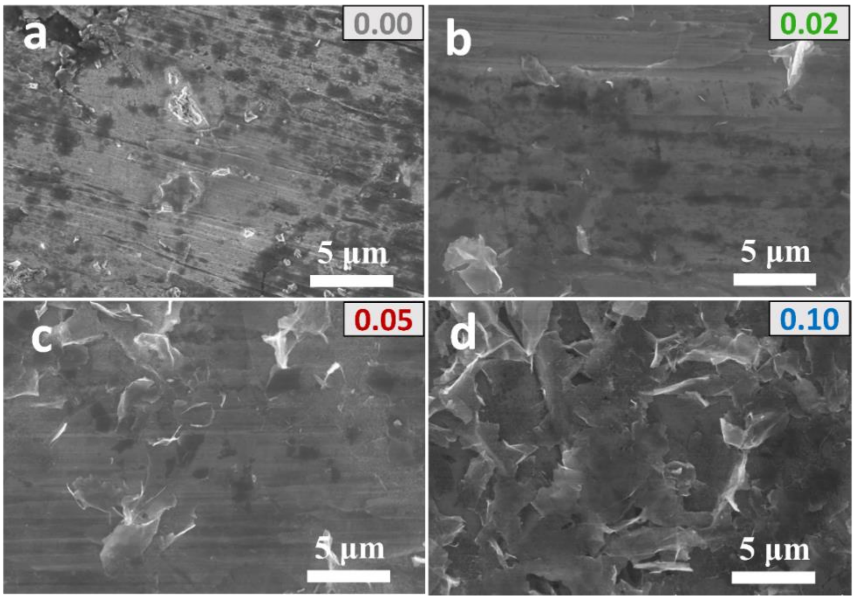

Fig. S6 Surface SEM images of Zn foil immersed in electrolyte with different concentration of MXene additives for 4 h: **a** 0 mg mL^{-1}, **b** 0.02 mg mL^{-1}, **c** 0.05 mg mL^{-1}, and **d** 0.10 mg mL^{-1}

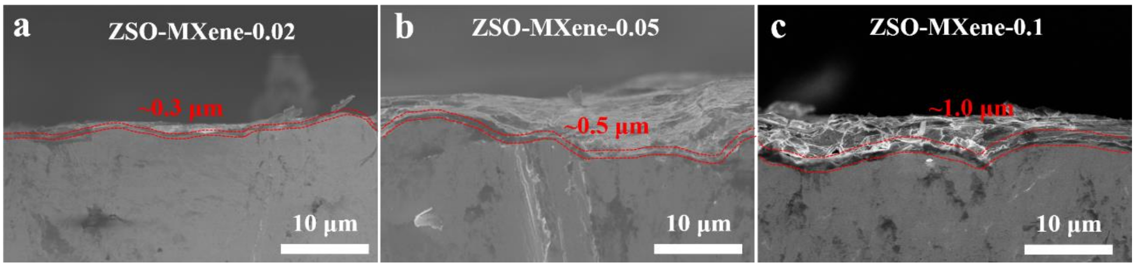

Fig. S7 Sectional SEM images of Zn foil immersed in electrolyte with different concentration of MXene additives for 4 h: **a** 0.02 mg mL^{-1}, **b** 0.05 mg mL^{-1}, and **c** 0.10 mg mL^{-1}

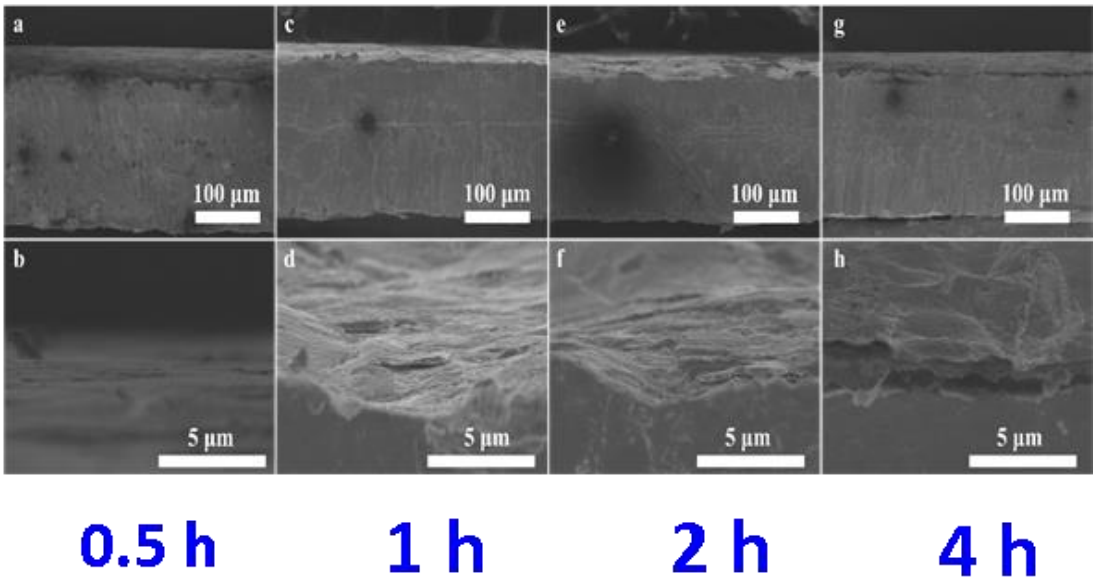

Fig. S8 Cross-sectional SEM imageS of Zn foil immersed in electrolyte with concentration of 0.05 mg mL^{-1} MXene additives for **a, b** 0.5 h, **c, d** 1 h, **e, f** 2 h, and **g, h** 4 h

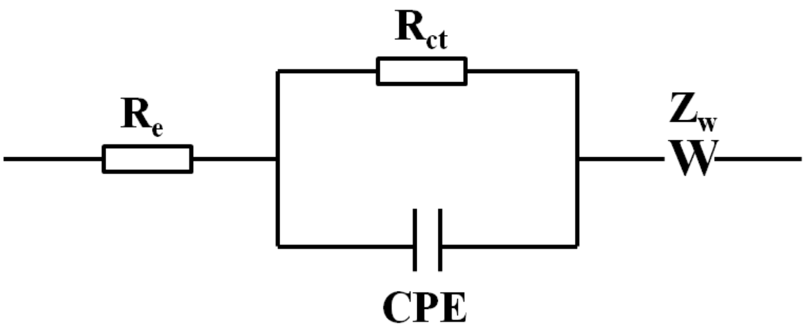

Fig. S9 The equivalent circuit

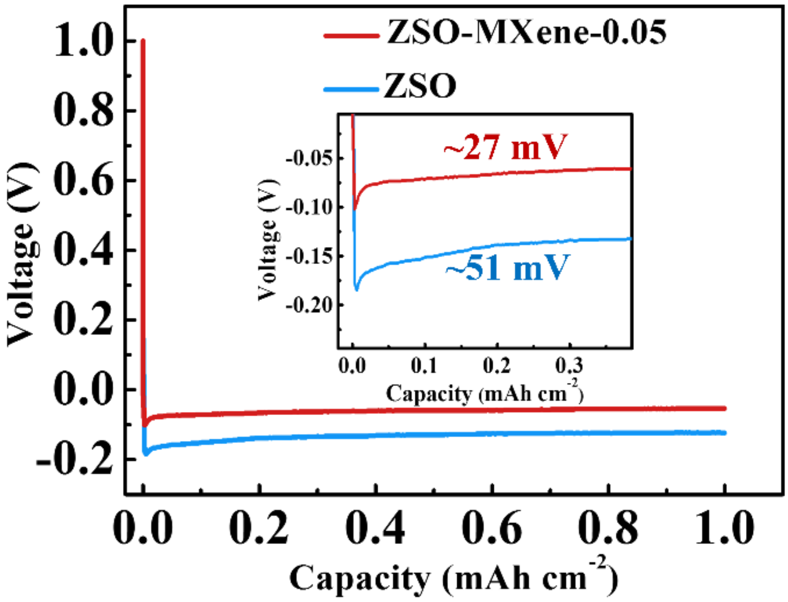

Fig. S10 Zn plating curves in electrolytes of ZSO-MXene-0.05 and ZSO at the current density of 2 mA cm^{-2} with the capacity limited to 1 mAh cm^{-2}

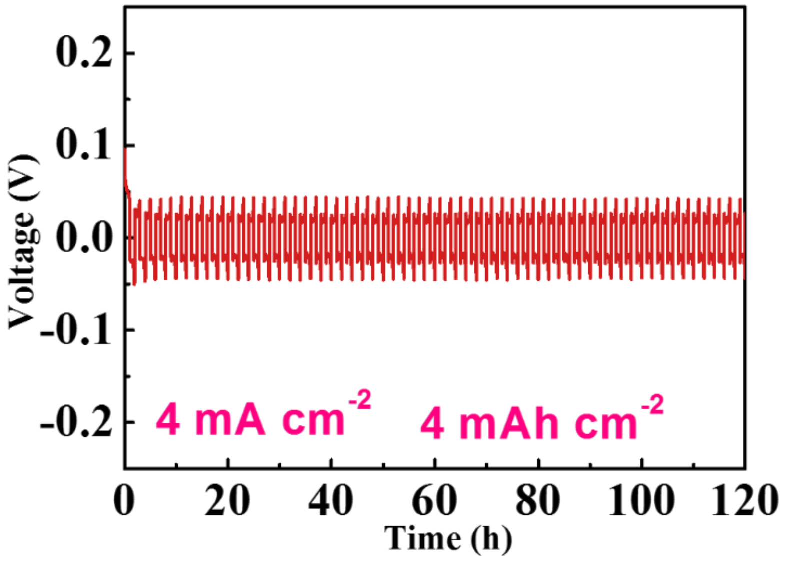

Fig. S11 Long-term galvanostatic cycling of Zn-Zn symmetrical cell at 4 mA cm^{-2} with 4 mAh cm^{-2}

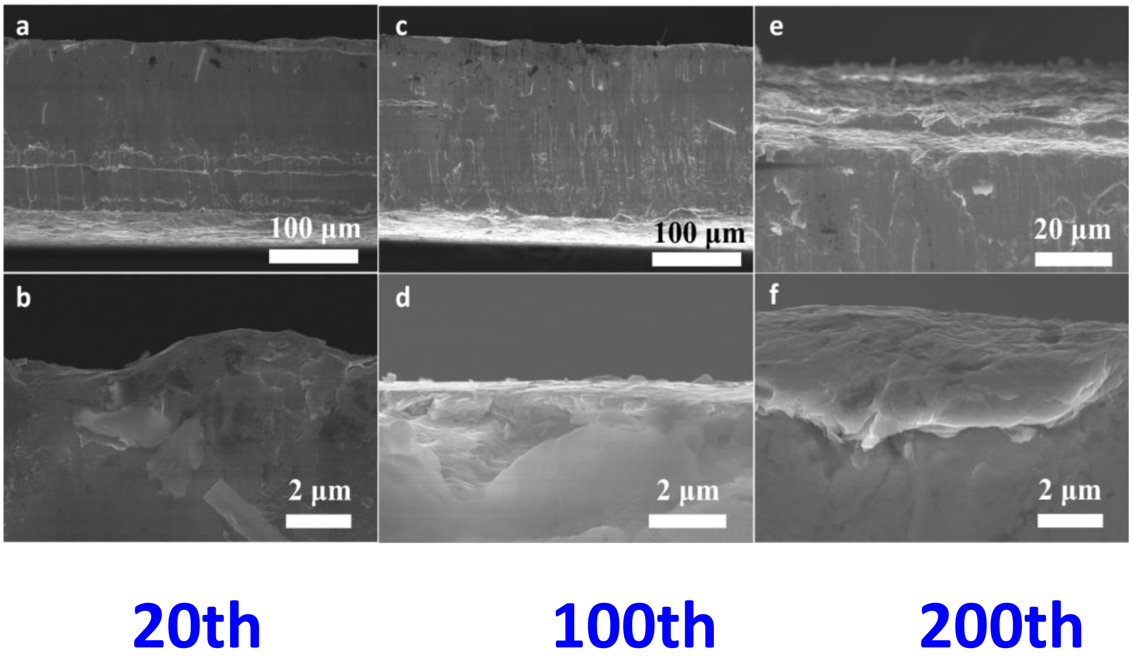

Fig. S12 The cross-sectional configuration of Zn anode after cycling in ZSO-MXene-0.05 electrolyte at current density of 2 mA cm^{-2} with 1 mAh cm^{-2} Zn plating/stripping: **a, b** after 20 cycles, **c, d** after 100 cycles and **e, f** after 200 cycles

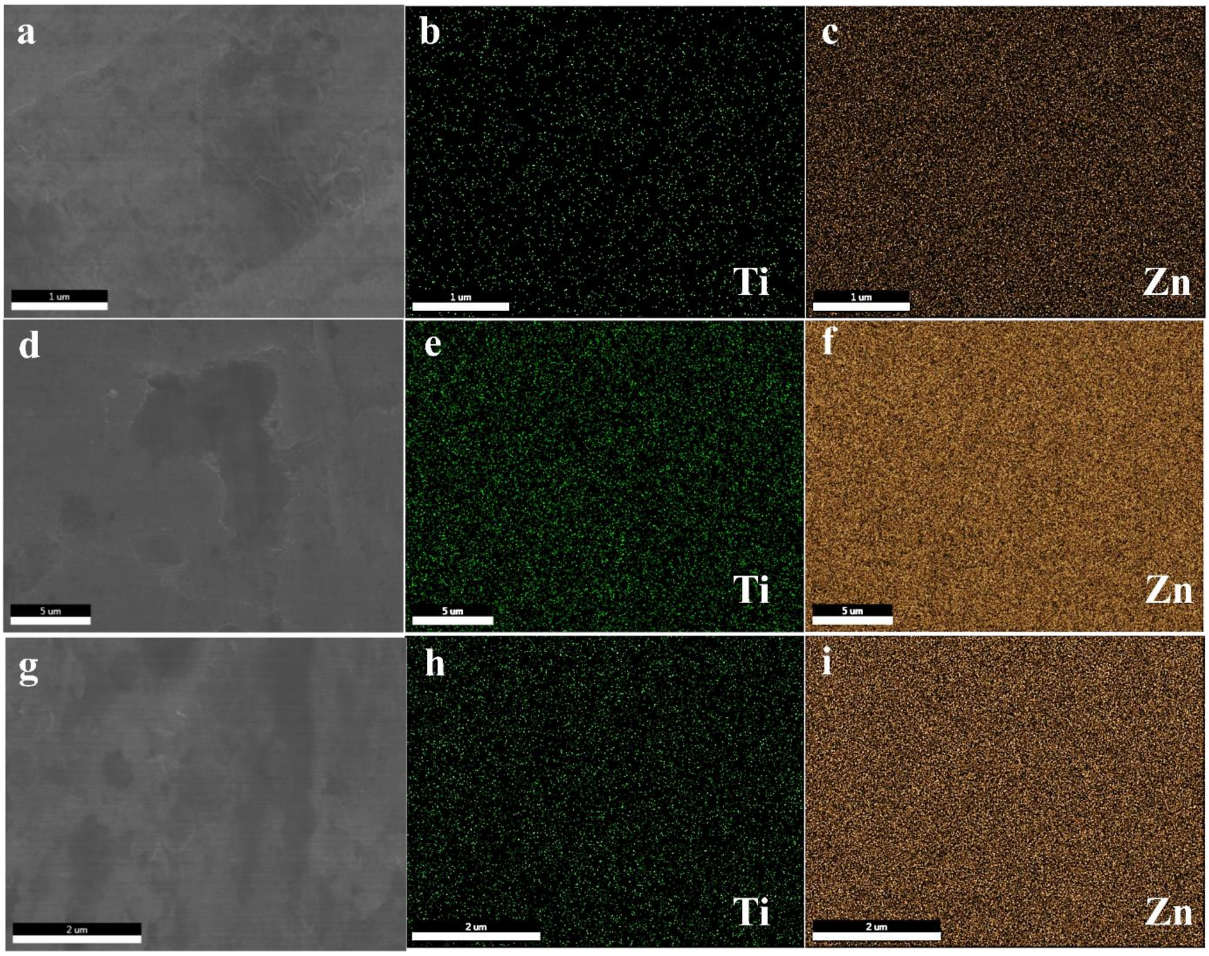

Fig. S13 Surface configuration of Zn anode after cycling in ZSO-MXene-0.05 electrolyte for different cycles and the corresponding EDS mapping results: **a-c** 20 cycles, **d-f** 100 cycles, and **g-i** 200 cycles

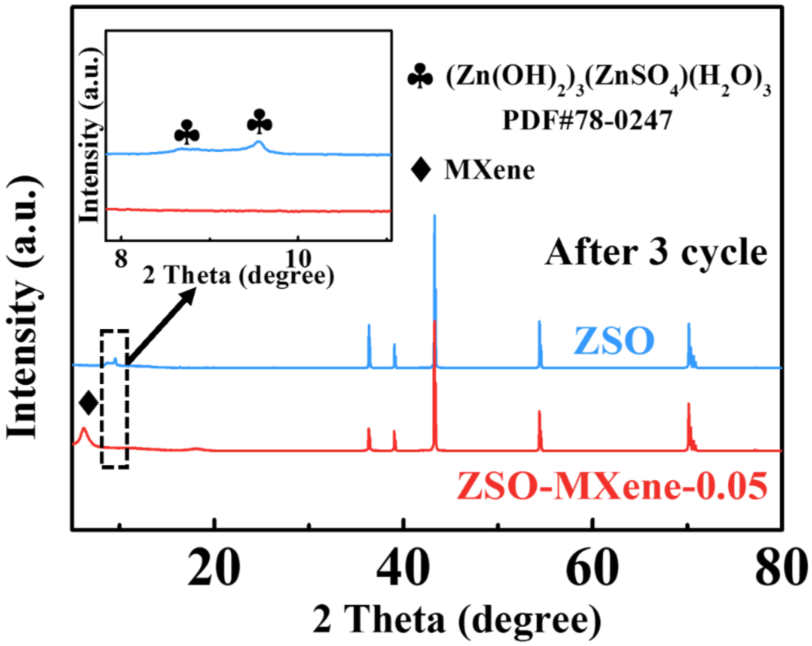

Fig. S14 XRD measurement of cycled Zn anode in the ZSO and ZSO-MXene-0.05 electrolytes

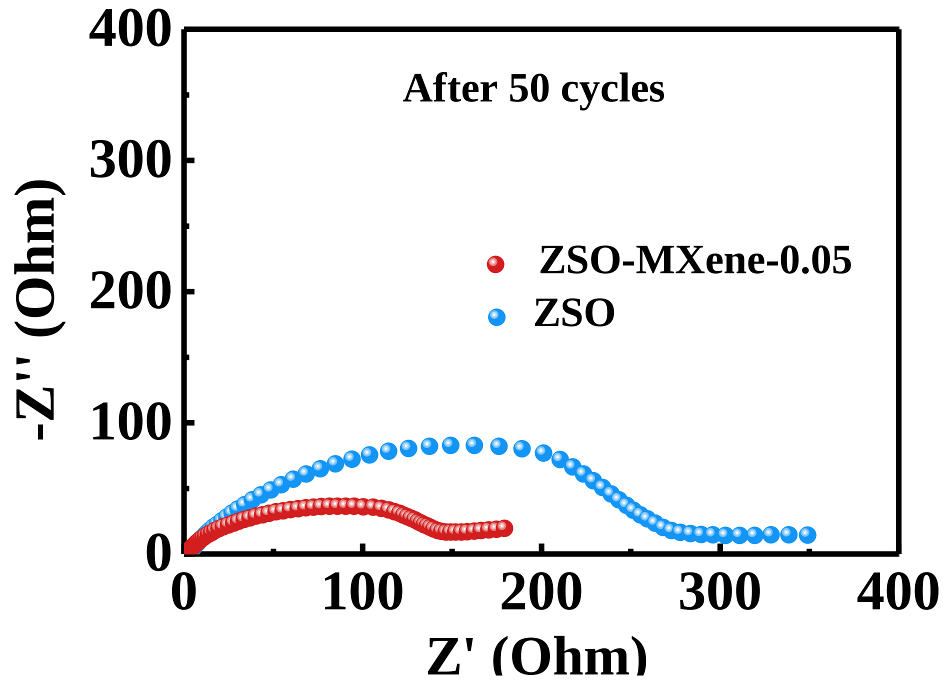

Fig. S15 EIS of Zn-Zn symmetrical cells in ZSO and ZSO-MXene-0.05 electrolyte after charge-discharged for 50 cycles

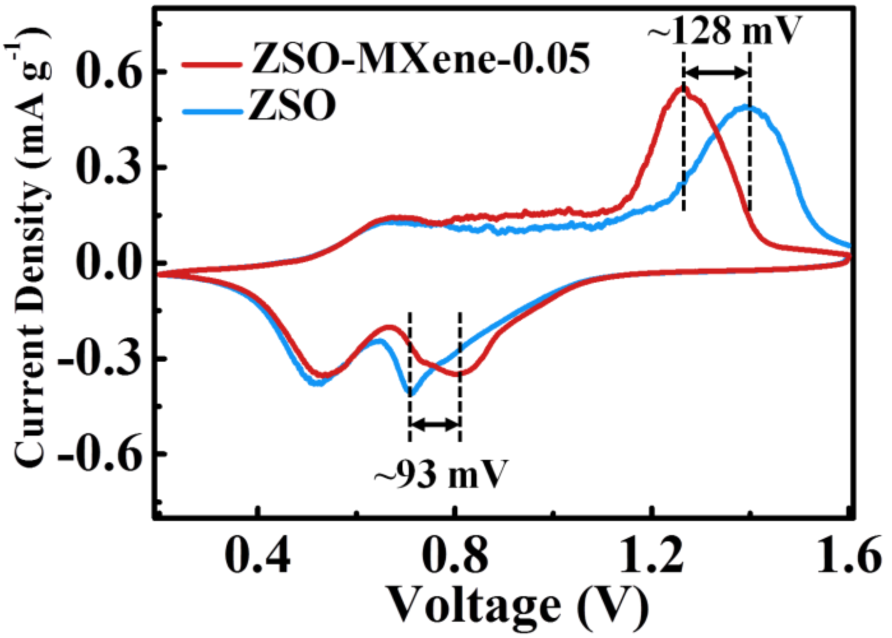

Fig. S16 CV curves of the Zn-V_2O_5 full cells in ZSO and ZSO-MXene-0.05 electrolyte

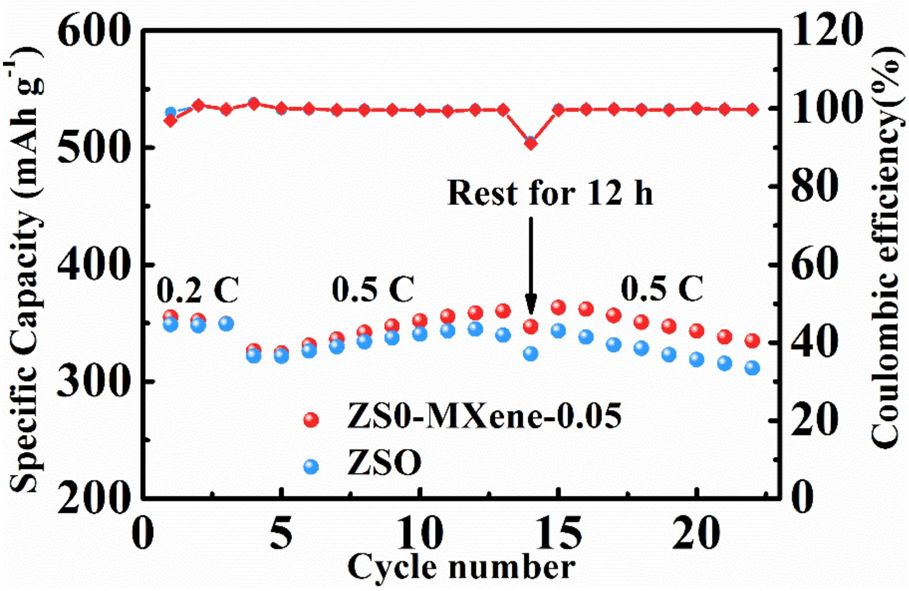

Fig. S17 Self-discharge behavior of Zn full cells

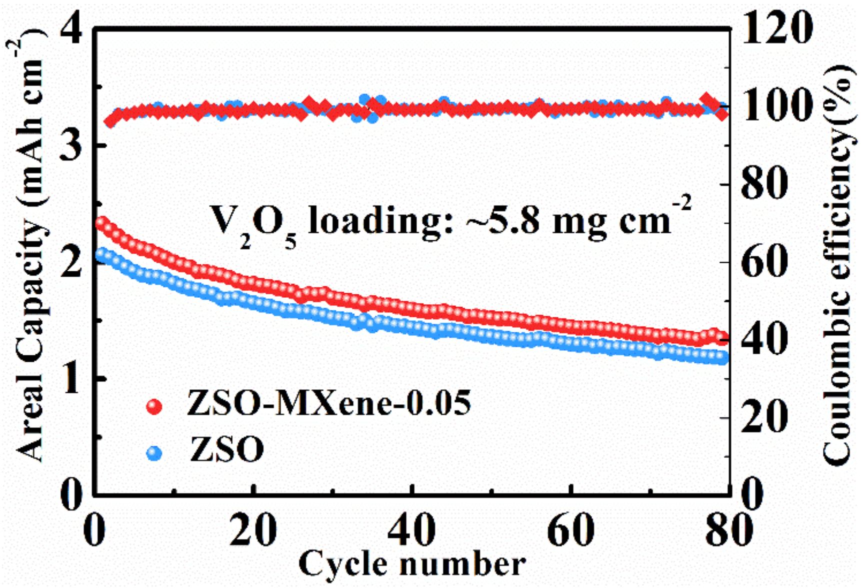

Fig. S18 Long-term cycling performance of Zn-V_2O_5 full cells in high mass loading

Table S1 Fitting results of the Nyquist plots of Zn-Zn cells in electrolytes with different MXene additives

Concentration	R_{ct} (Ω)
Blank $ZnSO_4$	1042.5
ZSO-MXene-0.02	725.3
ZSO-MXene-0.05	715.2
ZSO-MXene-0.10	767.9

Table S2 Cycling performances comparison for various Zn anodes

Strategies/Anode materials	Lifespan	Refs.
20 m LiTFSI+1 m Zn(TFSI)/Zinc power	170 h (0.2 mA cm^{-2}, 0.035 mAh cm^{-2})	*Nat. Mater.* **2018**, *17*, 543-549
3 M $Zn(CF_3SO_3)_2$/Zinc foil	800 h (0.1 mA cm^{-2}, 0.1mAh cm^{-2})	*J. Am. Chem. Soc.* **2016**, *138*, 12894
3.3 M $ZnSO_4$/MOF-coated Zn foils	1300 h (0.3 mA cm^{-2}, 0.3mAh cm^{-2})	*Angew. Chem.* **2020**, *59*, 9377
N-doped carbon coated zinc foil	400 h (2 mA cm^{-2}, 2 mAh cm^{-2})	*Adv. Energy Mater.* **2020**, *10*, 1904215
3D flexible carbon nanotubes	200 h (1 mA cm^{-2}, 2 mAh cm^{-2})	*Adv. Mater.* **2019**, *31*, 1903675.
$Ti_3C_2T_X$ MXene@Zn Paper	350 h (1 mA cm^{-2}, 1 mAh cm^{-2})	*ACS Nano* **2019**, *13*, 11676
PAM/Zinc plated copper mesh	350 h (0.2 mA cm^{-2}, 1 mAh cm^{-2})	*Angew. Chem.* **2019**, *58*, 15841.
Triethyl phosphate electrolyte/zinc foil	600 h (0.8 mA cm^{-2}, 0.8 mAh cm^{-2})	*Angew. Chem.* **2019**, *58*, 2760
Diethyl ether additive/zinc foil	250 h (0.2 mA cm^{-2}, 0.2 mAh cm^{-2})	*Nano Energy*. **2019**, *62*, 275
Nanoporous $CaCO_3$-coated zinc anode	836 h (0.25 mA cm^{-2}, 0.05 mAh cm^{-2})	*Adv. Energy Mater.* **2018**, *8*, 1801090.
**$Ti_3C_2T_X$ MXene additive/Zinc foil**	**500 h (1 mA cm^{-2}, 1 mAh cm^{-2})**<br>**1180 h (2 mA cm^{-2}, 1 mAh cm^{-2})**<br>**250 h (4 mA cm^{-2}, 1 mAh cm^{-2})**	**This work**

Table S3 Comparison for electrochemical performances of Zn-V_2O_5 cells

Cathode materials	Discharge capacity	Refs.
$V_2O_5·nH_2O$	381 mA h g^{-1} (60 mA g^{-1})	*Adv. Mater.* **2018**, *30*, 1703725
V_2O_5	242 mA h g^{-1} (50 mA g^{-1})	*Chem. Commun.* **2018**, *54*, 4457−4460.
V_2O_5	372 mA h g^{-1} (300 mA g^{-1})	*ACS Appl. Mater. Interfaces* **2017**, *9*, 42717−42722.
V_2O_5	196 mA h g^{-1} (14.4 mA g^{-1})	*Adv. Energy Mater*. **2016**, *6*, 1600826
V_2O_5	282 mA h g^{-1} (300 mA g^{-1})	*Nat Energy*. **2016**, *1*,16119
V_2O_5	340 mA h g^{-1} (200 mA g^{-1})	*Angew. Chem.* **2018**, 57, 3943.
V_2O_5	373 mA h g^{-1} (200 mA g^{-1})	*Adv. Energy Mater.* **2018**, *8*, 1702463
**V_2O_5**	**390.9 mA h g^{-1} (200 mA g^{-1})**	**This work**
